# Supplementary material for: Morphology and Molecular Phylogeny of Genus Oedogonium (Oedogoniales, Chlorophyta) from China
Source: Plants (Basel). 2022 Sep 16;11(18):2422. doi: 10.3390/plants11182422 (PMC9505714; doi:10.3390/plants11182422)
Supplement: Supplementary file 1 [file plants-11-02422-s001.zip › Supllementary figure S1 The microphotos of the eight morphological traits used to match with phylogenetic result.pdf]

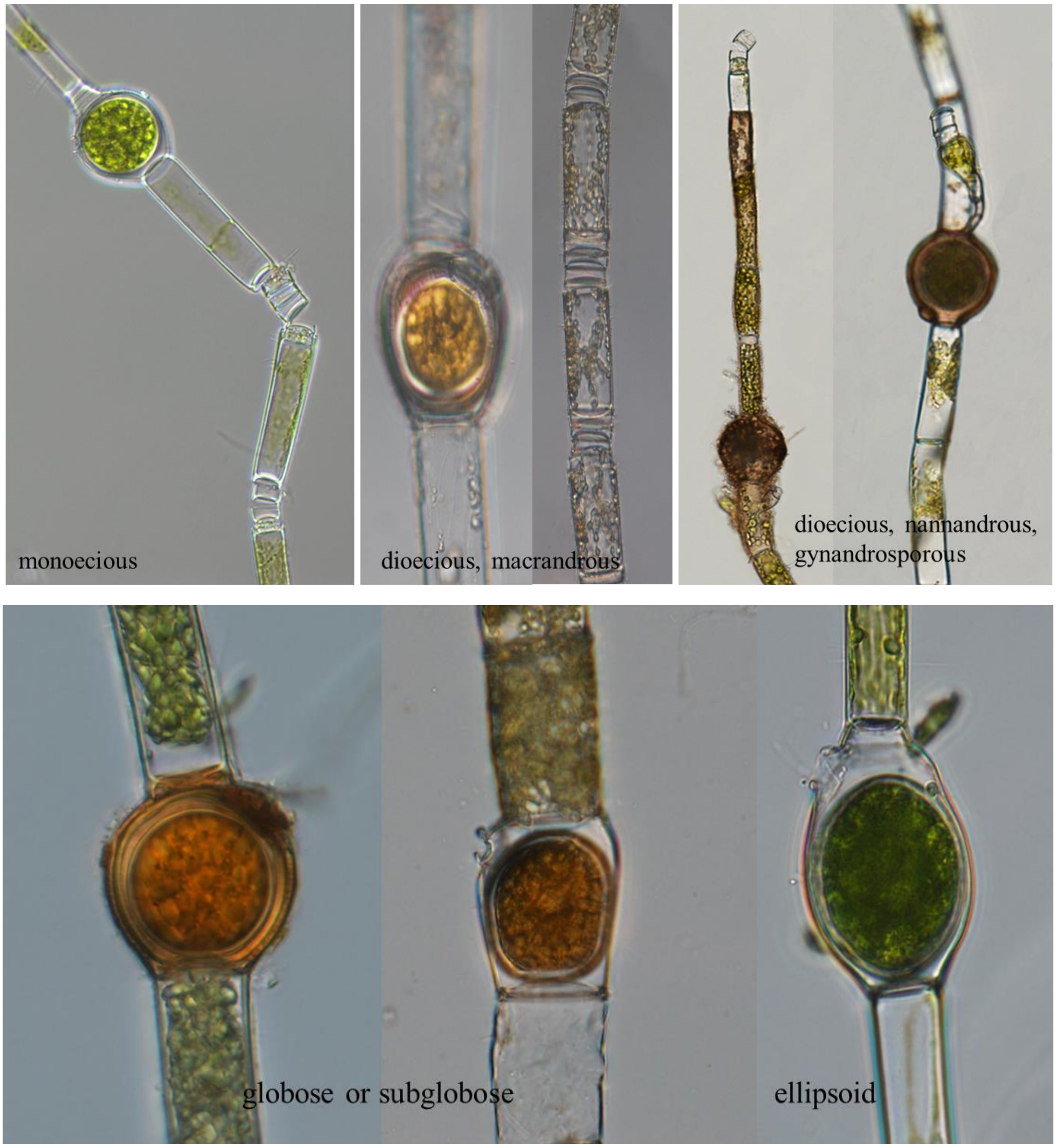
monoecious
dioecious, macrandrous
dioecious, nannandrous,
gynandrosporous
globose or subglobose
ellipsoid

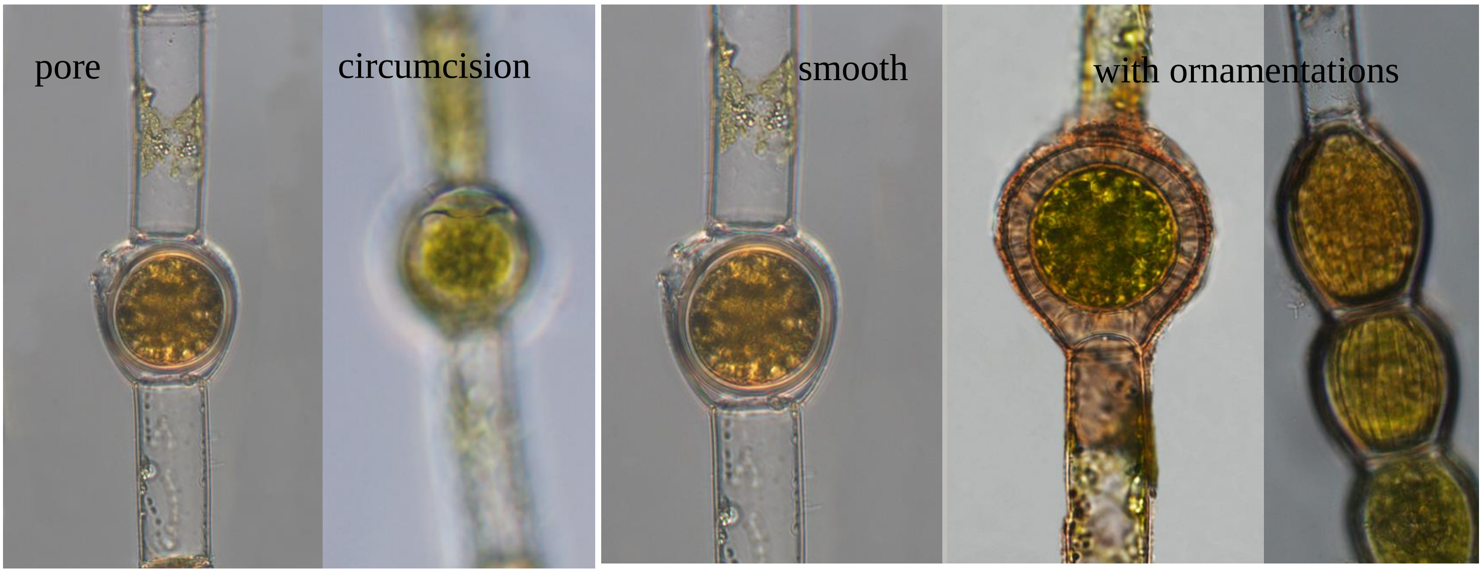
pore
circumcision
smooth
with ornamentations

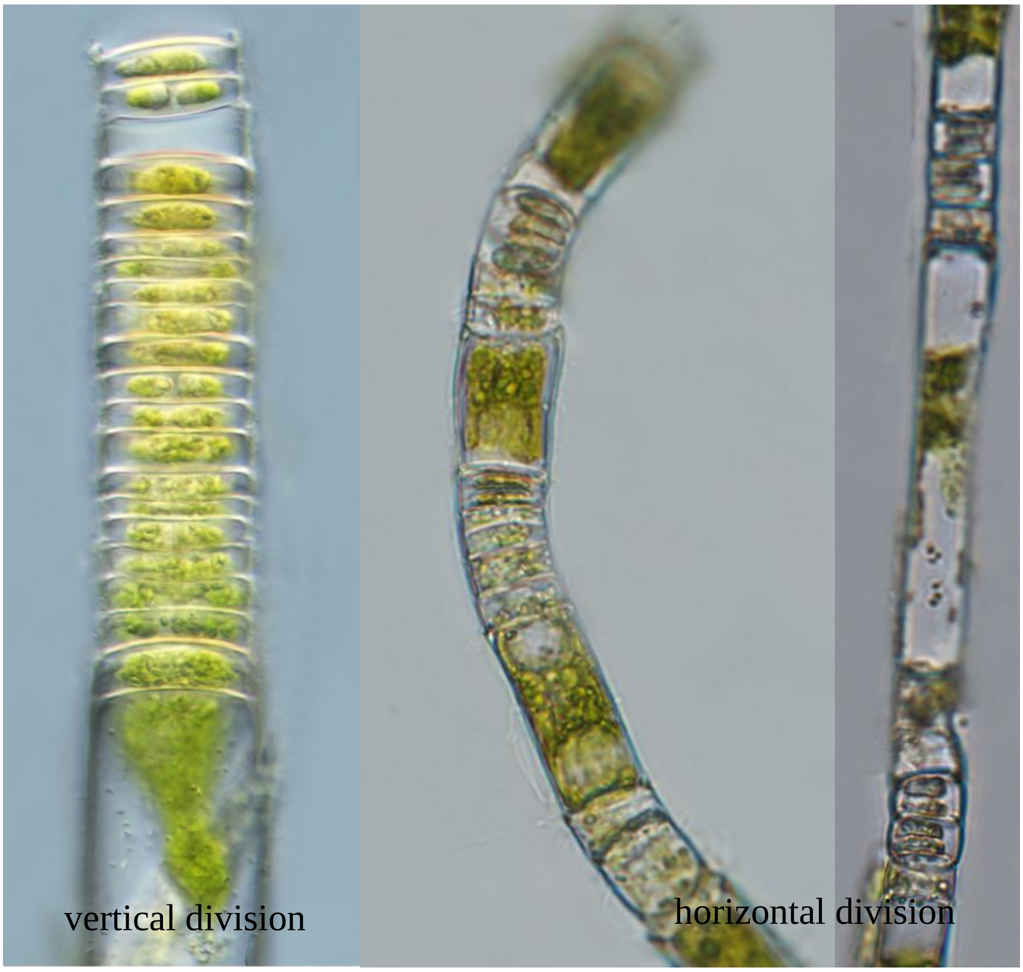
vertical division
horizontal division

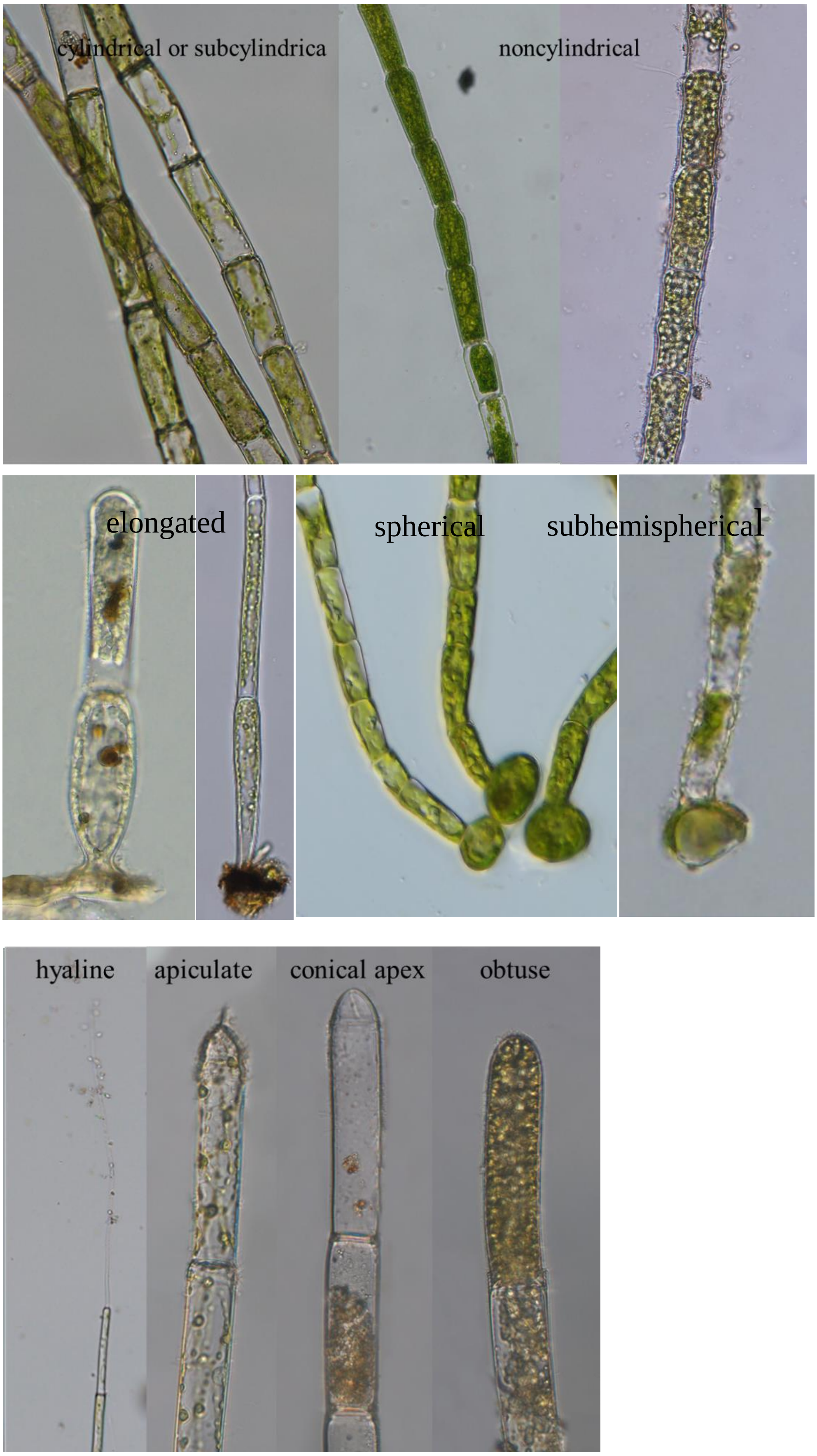
cylindrical or subcylindrica
noncylindrical
elongated
spherical
subhemispherical
hyaline
apiculate
conical apex
obtuse

Figure S1.
