# Supplementary material for: Morphology and Molecular Phylogeny of Genus Oedogonium (Oedogoniales, Chlorophyta) from China
Source: Plants (Basel). 2022 Sep 16;11(18):2422. doi: 10.3390/plants11182422 (PMC9505714; doi:10.3390/plants11182422)
Supplement: Supplementary file 1 [file plants-11-02422-s001.zip › Supplementary table S1 Location of the collecting sites and GenBank accession number of isolates.pdf]

Supplementary table S1. Location of the collecting sites and GenBank accession number of isolates.

Species name	Strain/Voucher no.	Locality	GenBank accession no.		
			18S rDNA	ITS	rbcl
Oedogonium sp.	BBG18524/FACHB-3502	Wuhan 30.54 N, 114.35 E	OM111213	OM111128	OM104041
Oedogonium sp.	CQ1912_8/FACHB-3503	Chongqing 29.84 N, 106.21 E	OM111212	OM111130	OM104045
Oedogonium sp.	CQ1913/FACHB-3504	Chongqing 29.84 N, 106.21 E	OM111211	OM111131	OM104046
Oedogonium sp.	CQ1916_3/FACHB-3505	Chongqing 29.84 N, 106.21 E	OM111210	OM111132	OM104047
Oedogonium sp.	CQ1921_5/FACHB-3506	Chongqing 29.84 N, 106.21 E	OM111209	OM111134	OM104049
Oedogonium sp.	CQ1923_5/FACHB-3507	Chongqing 29.84 N, 106.21 E	OM111208	OM111135	OM104050
Oedogonium sp.	CQ1923_8/FACHB-3508	Chongqing 29.84 N, 106.21 E	OM111207	OM111142	OM104051
Oedogonium sp.	CQ1931/FACHB-3509	Chongqing 29.84 N, 106.21 E	OM111206	OM111136	OM104053
Oedogonium sp.	CQ1932_4/FACHB-3510	Chongqing 29.84 N, 106.21 E	OM111205	OM111144	OM104054
Oedogonium sp.	CQ1933_1/FACHB-3511	Chongqing 29.84 N, 106.21 E	OM111204	OM111137	OM104055
Oedogonium sp.	CQ1940_1/FACHB-3512	Chongqing 29.84 N, 106.21 E	OM111203	OM111138	OM104056
Oedogonium sp.	CQ1940_2/FACHB-3513	Chongqing 29.84 N, 106.21 E	OM111202	OM111139	OM104057
Oedogonium sp.	FYT1801/FACHB-3514	Wuhan 30.54 N, 114.35 E	OM111201	OM111145	OM104058
Oedogonium sp.	HH1815_2/FACHB-3516	Huaihua 27.57 N, 110.04 E	OM111214	OM111141	OM104061
Oedogonium sp.	SD1809/FACHB-3515	Shandong 36.37 N, 120.24 E	OM111200	OM111152	OM104063
Oedogonium sp.	TS1907_1/FACHB-3518	Tongshan 29.43 N, 114.7 E	OM111198	OM111153	OM104065
Oedogonium sp.	TS1907_2/FACHB-3519	Tongshan 29.43 N, 114.7 E	OM111197	OM111154	OM104066
Oedogonium sp.	TS1907_4/FACHB-3520	Tongshan 29.43 N, 114.7 E	OM111196	OM111155	OM104067
Oedogonium varians	TS191012/FACHB-3517	Tongshan 29.43 N, 114.7 E	OM111199	OM111157	OM104069
Oedogonium sp.	WH1913_6/FACHB-3522	Wuhan 30.5 N, 114 E	OM111195	OM111161	OM104074
Oedogonium sp.	WH1914_1/FACHB-3523	Wuhan 30.5 N, 114 E	OM111194	OM111162	OM104075
Oedogonium sp.	WH1920_1/FACHB-3524	Wuhan 30.5 N, 114 E	OM111193	OM111163	OM104076

Oedogonium sp.	WH1924_2/FACHB-3526	Wuhan 30.5 N, 114 E	OM111192	OM111165	OM104078
Oedogonium sp.	WH1924_3/FACHB-3527	Wuhan 30.5 N, 114 E	OM111191	OM111166	OM104079
Oedogonium mirificum	WH1931_10/FACHB-3528	Wuhan 30.5 N, 114 E	OM111190	OM111167	OM104080
Oedogonium mirificum	WH1931_11/FACHB-3529	Wuhan 30.5 N, 114 E	OM111189	OM111168	OM104081
Oedogonium mirificum	WH1931_6/FACHB-3530	Wuhan 30.5 N, 114 E	OM111188	OM111169	OM104082
Oedogonium cyathigerum	WH1931_8/FACHB-3531	Wuhan 30.5 N, 114 E	OM111187	OM111148	OM104083
Oedogonium sp.	WLQ1851010_1/FACHB-3532	Wuhan 30.5 N, 114 E	OM111186	OM111171	OM104085
Oedogonium sp.	WLQ1852911_4_4/FACHB-3533	Wuhan 30.5 N, 114 E	OM111185	OM111172	OM104086
Oedogonium capillare	XQ1802/FACHB-3534	Wuhan 30.5 N, 114 E	OM111184	OM111149	OM104087
Oedogonium sp.	XQ1804/FACHB-3535	Wuhan 30.5 N, 114 E	OM111183	OM111150	OM104088
Oedogonium sp.	XT1902_1/FACHB-3536	Xiantao 30.4 N, 113.16 E	OM111182	OM111174	OM104091
Oedogonium sp.	XT1902_3/FACHB-3537	Xiantao 30.4 N, 113.15 E	OM111181	OM111175	OM104092
Oedogonium sp.	XT1902_5/FACHB-3538	Xiantao 30.4 N, 113.14 E	OM111180	OM111176	OM104093
Oedogonium crispum	CQ05/FACHB-3310	Jaocheng 37.57 N, 114.40 E		OM111129	OM104044
Oedogonium sp.	CQ1916_4/FACHB-3539	Chongqing 29.84 N, 106.21 E		OM111133	OM104048
Oedogonium dentireticulatum	CQ1925_1/FACHB-3309	Chongqing 29.84 N, 106.21 E		OM111143	OM104052
Oedogonium sp.	GDQY3/FACHB-3540	Qingyuan 23.75 N, 113.16 E		OM111146	OM104059
Oedogonium sp.	HH1810_2/FACHB-3541	Huaihua 27.57 N, 110.04 E		OM111140	OM104060
Oedogonium obpyriforme	TS1910_7/FACHB-3542	Tongshan 29.43 N, 114.7 E		OM111156	OM104068
Oedogonium sp.	WD1801/FACHB-3521	Wuhan 30.55 N, 114.36 E		OM111158	OM104070
Oedogonium capilliforme	WH1904/FACHB-3312	Wuhan 30.5 N, 114 E		OM111147	OM104071
Oedogonium sp.	WH1911_4/FACHB-3543	Wuhan 30.5 N, 114 E		OM111159	OM104072
Oedogonium sp.	WH1913_5/FACHB-3544	Wuhan 30.5 N, 114 E		OM111160	OM104073
Oedogonium crispum var. *hawaiense*	WH1924_1/FACHB-3317	Wuhan 30.5 N, 114 E		OM111164	OM104077
Oedogonium sp.	WLQ1851006_1_2/FACHB-3545	Wuhan 30.5 N, 114 E		OM111170	OM104084

Oedogonium sp.	XQ1819/FACHB-3546	Wuhan 30.5 N, 114 E	OM111151	OM104089
Oedogonium sp.	XQ1825/FACHB-3547	Ningbo 29.76N,121.35 E	OM111173	OM104090
Oedogonium sp.	ZWY1801_6/FACHB-3548	Wuhan 30.5 N,114.42 E	OM111177	OM104094
Oedogonium sp.	ZWY1805/FACHB-3549	Wuhan 30.5 N,114.42 E	OM111178	OM104095
Oedogonium sp.	ZWY1903_1/FACHB-3311	Wuhan 30.5 N,114.42 E	OM111179	OM104096
Oedocladium prescottii	BN3/FACHB-2452	Xishuangbanna 21.92 N, 101.26 E		OM104042
Oedocladium carolinianum	BN4/FACHB-2455	Xishuangbanna 21.92 N, 101.26 E		OM104043
Oedocladium carolinianum	OE_X8/FACHB-2456	Xiantao 30.4 N, 113.16 E		OM104062
Oedocladium carolinianum	TB19/FACHB-2453	Tibet 29.81 N, 93.77 E		OM104064
