# Supplementary material for: Morphology and Molecular Phylogeny of Genus Oedogonium (Oedogoniales, Chlorophyta) from China
Source: Plants (Basel). 2022 Sep 16;11(18):2422. doi: 10.3390/plants11182422 (PMC9505714; doi:10.3390/plants11182422)
Supplement: Supplementary file 1 [file plants-11-02422-s001.zip › Supplementary table S2 Strains whose sequences were downloaded from GenBank.pdf]

Supplementary table S2. Strains whose sequences (18S, ITS and rbcl) were downloaded from GenBank.

18S rDNA		ITS		rbcl	
Taxon	GenBank accession no.	Taxon	GenBank accession no.	Taxon	GenBank accession no.
Oedogonium nodulosum	DQ018735	*Oedogonium borisianum*	AY962670	*Oedogonium nodulosum*	DQ481204
Oedogonium subplagiostomum	DQ078295	*Oedogonium calliandrum*	AY962672	*Oedogonium subplagiostomum*	DQ481205
Oedogonium tenerum	DQ078296	*Oedogonium cardiacum*	AY962675	*Oedogonium subplagiostomum*	DQ481205
Oedogonium cylindrosporum	DQ078297	*Oedogonium angustistomum*	AY962676	*Oedogonium* sp.	DQ481206
Oedocladium prescottii	DQ078298	*Bulbochaete rectangularis* var. *hiloensis*	AY962677	*Oedogonium* sp.	DQ481207
Oedogonium brevicingulatum	DQ078299	*Oedogonium vaucherii*	AY962678	*Oedogonium subdissimile*	DQ481208
Oedogonium acrosporum	DQ115892	*Oedogonium fragile*	AY962679	*Oedogonium* sp.	DQ481209
Oedogonium pusillum	DQ115898	*Oedogonium crispum*	AY962680	*Oedogonium* sp.	DQ481210
Oedogonium pluviale	DQ115899	*Oedogonium oblongum*	AY962681	*Oedogonium brevicingulatum*	DQ481211
Oedogonium sp.	DQ413048	*Oedogonium cylindrosporum*	DQ078300	*Oedocladium prescottii*	DQ481212
Oedogonium sp.	DQ413049	*Oedogonium nodulosum*	DQ078301	*Oedogonium* sp.	DQ481213
Oedogonium sp.	DQ413050	*Oedogonium eminens*	DQ078302	*Oedogonium pakistanense*	DQ481214
Oedogonium sp.	DQ413051	*Oedogonium tenerum*	DQ178024	*Oedogonium* sp.	DQ481215
Oedogonium brevicingulatum	DQ413052	*Oedogonium undulatum*	DQ178025	*Bulbochaete rectangularis* var. *hiloensis*	EF113415
Oedogonium sp.	DQ413053	*Oedogonium subplagiostomum*	DQ413054	*Oedogonium cardiacum*	EF113458
Oedogonium sp.	DQ418462	*Oedogonium brevicingulatum*	DQ413058	*Oedogonium cardiacum*	EF587355
Oedogonium howardii	EF616486	*Oedogonium pakistanense*	DQ413060	*Oedogonium cardiacum*	EF587355
Oedogonium cardiacum	EU123943	*Oedocladium prescottii*	DQ450898	*Oedogonium cardiacum*	EF589157

Bulbochaete hiloensis	U83132	*Oedogonium subplagiostomum*	KY575148	*Oedocladium carolinianum*	JQ394813
Oedogonium cardiacum	U83133	*Oedogonium* sp.	DQ413055	*Oedogonium angustistomum*	KT693218
Oedogonium angustistomum	U83134	*Oedogonium* sp.	DQ413056	*Stigeoclonium helveticum*	DQ630521
Oedocladium carolinianum	U83135	*Oedogonium* sp.	DQ413057	*Schizomeris leibleinii*	HQ700713
Oedogonium capilliforme	MZ322844	*Oedogonium* sp.	DQ413059		
Oedogonium crispum var. *hawaiense*	MZ457075	*Oedogonium* sp.	DQ178023		
Oedogonium sp.	MZ322845	*Oedocladium prescottii*	MT431672		
Oedogonium crispum	MZ322846	*Oedocladium carolinianum*	MT539289		
Oedogonium dentireticulatum	MZ322843	*Oedocladium carolinianum*	MT539290		
Oedocladium prescottii	MT431686	*Oedocladium carolinianum*	MT431673		
Oedocladium carolinianum	MT539287	*Schizomeris leibleinii*	HQ646381		
Oedocladium carolinianum	MT539288	*Stigeoclonium helveticum*	HQ646382		
Oedocladium carolinianum	MT431687				
Stigeoclonium helveticum	EU123941				
Schizomeris leibleinii	AF182820				
